# PREVALENCE AND MANAGEMENT OF HYPERTENSION AMONG MIDDLE-AGED AND OLDER ADULTS IN CHINA

**DOI:** 10.1093/geroni/igz038.451

**Published:** 2019-11-08

**Authors:** Jianjia Cheng, Jungtae Choi, Lydia Li

**Affiliations:** 1 School of Social Work, University of Michigan, Ann Arbor, Michigan, United States; 2 University of Michigan, Ann Arbor, Michigan, United States

## Abstract

The study aimed to examine hypertension prevalence and management in China. Data were from a national survey of a probability sample of 45+ in China (N = 17,047). Self-reported hypertension diagnosis and readings from electronic blood pressure monitors were used to create four variables: diagnosed, measured (>=140/90 as high), undiagnosed, and overall (diagnosed + undiagnosed) hypertension. Respondents with diagnosed hypertension were asked about medication use, blood pressure monitoring, and lifestyle advice from doctors; and were considered inadequate blood pressure control if having measured hypertension. Weighted descriptive statistics and multivariate logistic regression were conducted. The prevalence of diagnosed, measured, undiagnosed and overall hypertension was 27%, 37%, 14%, and 51%, respectively. Across all four, older age adults, women, and urban residents had higher rates. Among hypertensive patients, 82% took anti-hypertensive medications, 91% monitored blood pressure, 60% received lifestyle advice, and 53% had inadequate blood pressure control. Compared to the 45-54 years old, the 75+ was less likely to receive lifestyle advice (OR=0.63, 95% CI=0.43-0.95) and the 65-74 was less likely to have adequate control (OR=0.75, 95% CI=0.73-0.98). Men were less likely to use medications (OR=0.77, 95% CI=0.63-0.95) but more likely to receive lifestyle advice (OR=1.48, 95% CI=1.23-1.78) than women. More education and urban (vs. rural) were associated with better hypertension management and control. In conclusion, hypertension affects half of the middle-aged and older population in China. More than half of hypertensive patients have inadequate blood pressure control. People who are older, women, low-educated, and rural residents are disadvantaged in hypertension management.

